# Antibody Response After Initial Vaccination for SARS-CoV-2 in Patients With Amyloidosis

**DOI:** 10.1097/HS9.0000000000000614

**Published:** 2021-07-15

**Authors:** Efstathios Kastritis, Evangelos Terpos, Aimilia Sklirou, Foteini Theodorakakou, Despina Fotiou, Eleni-Dimitra Papanagnou, Tina Bagratuni, Nikolaos Kanellias, Maria Gavriatopoulou, Ioannis P. Trougakos, Meletios A. Dimopoulos

**Affiliations:** 1 Department of Clinical Therapeutics, National and Kapodistrian University of Athens, Greece; 2 Department of Cell Biology and Biophysics, Faculty of Biology, National and Kapodistrian University of Athens, Greece

In systemic amyloidoses, misfolded proteins form amyloid fibrils that are deposited in various tissues causing organ dysfunction. In systemic immunoglobulin light chain (AL) amyloidosis, a usually small plasma cell clone produces a monoclonal immunoglobulin light chain with amyloidogenic properties. Although modest in size, this plasma cell clone may affect immunocompetence, while organ dysfunction further increases susceptibility and risk of complications of infectious diseases. In addition, the use of anti-plasma cell therapies further decreases the fitness of the immune system. In other types of amyloidosis, most common being transthyretin-related amyloidosis (ATTR), this immunosuppressive effect may not be present but other factors related to organ dysfunction may affect the fitness of the immune system. Thus, COVID-19 is particularly challenging for patients with critical organ involvement by amyloidosis.^1,2^ Vaccination against SARS-CoV-2 is the best strategy to avoid severe COVID-19, however, response to vaccines may be compromised in patients with plasma cell malignancies or other B-cell lymphoproliferative disorders.^3,4^ In patients with ATTR such a clone does not exist, but other factors could affect response to vaccination. Since it is clinically relevant to evaluate the efficacy of anti-SARS-CoV-2 vaccination in patients with AL and ATTR amyloidosis, to optimize strategies for the immunization of this vulnerable patient population, we measured the titers of neutralizing antibodies (NAbs) against SARS-CoV-2 after the first dose of the BNT162b2 and AZD1222 vaccines.

This report is part of larger prospective study (NCT04743388) for the kinetics of anti-SARS-CoV-2 antibodies after COVID-19 vaccination. The major inclusion criteria for this analysis include: (1) a prior diagnosis of AL or ATTR amyloidosis; (2) eligibility for vaccination. As a control group we used volunteers matched for age, gender (1:3) who had (1) no autoimmune or active malignant disease; (2) no HIV or active hepatitis B and C infection. Serum was separated within 4 hours from blood collection and stored at −80°C until the day of measurement on (1) day 1 (D1; before the first dose of BNT162b2 or AZD1222) and (2) day 22 (D22; before the second dose of the BNT162b2 or 3 weeks after the first dose of AZD1222). NAbs against SARS-CoV-2 were measured using FDA-approved methodology (ELISA, cPass SARS-CoV-2 NAbs Detection Kit; GenScript, Piscataway, NJ).^5^ The study was approved by the respective Ethical Committees in accordance with the Declaration of Helsinki and all patients and controls provided written informed consent before enrollment.

Our study included 59 patients with AL amyloidosis (34 males/25 females; median age: 62, interquartile range [IQR]: 57–72 years) and 118 controls (68 males/50 females; median age: 62, IQR: 57–72 years), who were vaccinated during the same period, at Alexandra Hospital, Athens, Greece. The patients followed a centrally controlled vaccination program run by the Greek healthcare authorities that prioritized healthcare workers, the elderly and patients with malignancies for anti-SARS-CoV-2 vaccination (with either BNT162b2 or AZD1222). The proportion of the different vaccines was similar in patient and control groups (78% had BNT162b2 and 22% had AZD1222, respectively). At the time of vaccination, 31/59 (53%) patients were receiving anti-clonal therapy. Table 1 shows the other characteristics of the patients.

**Table 1.** Characteristics of the Patients With AL Amyloidosis Who Were Included in the Analysis.

Male/female	34 (58%)/25 (42%)
Age, median (range)	62 (35–86)
BNT162b2	46(78%)
AZD1222	13 (22%)
Renal involvement	41 (69%)
Heart involvement	38 (64%)
Liver involvement	7 (12%)
PNS/ANS involvement	11(19%)
Mayo stage 1/2/3	22%/51% /27%
Bone marrow plasma cells at diagnosis	10% (0–30%)
eGFR (mL/min/1.73 m^2^)	77 (9–>150)
On active treatment at the time of vaccination	31 (53%)
Therapies (prior/current)	
Bortezomib or Ixazomib	41 (69%)/13 (22%)
Daratumumab	16 (27%)/11 (19%)
Lenalidomide	6(10%)/3 (9%)
Cyclophsophamide	37 (63%)/13 (22%)
Prior ASCT	4 (7%)
Disease in hematologic remission (CR or VGPR)	44 (75%)

ANS = autonomic nervous system; AL = immunoglobulin light chain; ASCT = autologous stem cell transplantation; CR = complete response; eGFR = estimated glomerular filtration rate; PNS = peripheral nervous system; VGPR = very good partial response.

At baseline, before the first vaccine shot (ie, on D1), only 5 (8%) patients and 14 (12%) controls had NAb titers of ≥30% (positivity cutoff) and there was no significant difference of the NAb titers between patients and controls (median 11.95% versus 15.2%, *P* = 0.831). Four patients with AL had baseline NAb titers above 50%, of which only 2 reported a history of COVID-19 infection.

On day 22 after the first dose, there was a significant increase in the levels of NAbs in controls (paired t-test, *P* < 0.001) as well as in those with AL amyloidosis (paired t-test, *P* = 0.0027); however, the median NAb titers for AL patients was 22.9% (range 0.5%–97.98%) compared to 44.9% (range 1.8%–98.3%) in controls (*P* < 0.001), so that 25/59 (42.4%) versus 78.5% developed NAb titers ≥30% (*P* < 0.001) (Figure 1A) and 14/59 (23.7%) AL patients versus 44% controls (*P* = 0.008) developed NAb titers ≥50% (which is the clinically relevant threshold for viral inhibition^6^). NAbs on D22 were similar for BNT162b2 and AZD1222.

**Figure 1.**
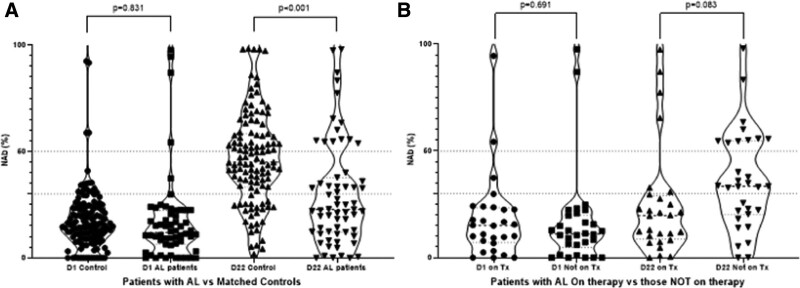
(A) Kinetics of neutralizing antibodies (NAbs) in patients with AL amyloidosis and age and sex-matched controls before and after the first vaccination dose for SARS-CoV-2 (with BNT162b2 or AZD1222 vaccine). (B) Kinetics of neutralizing antibodies (NAbs) in patients with AL amyloidosis on active therapy vs those not currently receiving anti-clonal therapy. AL = immunoglobulin light chain.

Among the 14 patients with AL amyloidosis and NAbs >50% at D22, 12 received BNT162b2, and 2 received AZD1222, 11/14 had clonal disease in deep remission (hematologic complete response or very good partial response) and 4 patients were still on treatment: 1 with ixazomib/dexamethasone, 1 with lenalidomide/dexamethasone, 1 with VCD (and prior history of COVID), and 1 with daratumumab. Among the other 10 patients, 7 had prior treatment with daratumumab in their most recent line of therapy that was discontinued at least 6 months ago (1 had also a prior history of COVID-19 infection). We also compared NAb titers among those on therapy and those being at least 3 months post-therapy: median Nab titers were 33.4% (range 14.3–55.7) versus 19.8% (range 0.5%–77.4%) (*P* = 0.083) (Figure 1B). We found no significant differences in NAbs on D22 among those with or without renal, cardiac, PNS/ANS, or liver involvement.

We also compared NAbs titers of patients with ATTRwt (N = 14) to age- and gender-matched controls (N = 28, 1:2 matching, median age 83, range 72–92 in both): median baseline (24% versus 14%) and D22 (39% versus 22%) NAbs were not significantly different. All were vaccinated with BNT162b2.

To the best of our knowledge, this is the first data on the humoral response after vaccination for SARS-CoV-2 in patients with AL and ATTR amyloidosis. Compared to a matched control population, patients with AL amyloidosis develop lower level of NAbs, corresponding to a less intense response of their adaptive humoral immunity, despite the low tumor burden. The immunosuppressive role of the underlying plasma cell clone in patients with AL amyloidosis (although tumor burden is low) is critical and these results are not significantly different from data in patients with myeloma (published from our group^3^ and others^7^). Patients with amyloidosis but no clonal disease (ie, those with ATTR) developed NAbs response similar to their matched controls. We also observed that anti-clonal therapy during the period of vaccination may affect humoral response. Although it is recommended that a treatment-free period should precede and follow vaccination, this may not be feasible: vaccination is an urgent need for those most vulnerable but, it may not be possible to hold treatment for a disease like AL amyloidosis. Nevertheless, patients on therapy who are in deep hematologic remission may still mount adequate NAbs titers. Interestingly, 4 “responders” had prior use of daratumumab, an anti-CD38 monoclonal antibody targeting the plasma cell compartment. However, to optimize immunization strategies, the timing of vaccination and the role of concomitant therapy requires further investigation. At this point, with still a limited number of patients, we found no correlations between patient characteristics and response. The impact of therapy or a specific therapy needs to be further evaluated and may change with larger numbers of patients or after the second dose or after longer follow up. Importantly, although the numbers are small, NAbs responses are similar for both BNT162b2 and AZD1222.

A significant proportion of the control group did not develop Nabs >50% at D22, but it is known that after the second dose (at least for BNT162b2) a substantial proportion develops very high titers, even among the elderly.^8^ Responses were also less robust in the very elderly, which includes most patients with ATTRwt. Thus, a second vaccine dose should always be given in patients with AL amyloidosis (and probably other lymphoproliferative disorders or with immunosuppression) as well as the elderly^8^ to increase the probability of adequate humoral response to vaccination.

In conclusion, patients with AL amyloidosis have a less robust response to anti-SARS-CoV-2 vaccination after the first dose compared to matched controls; a second dose is necessary and perhaps additional booster doses may be needed (or use of different vaccine) in those not mounting an adequate humoral response.

## Acknowledgments

We thank Ioanna Charitaki, RN; Christine Ivy Liacos, MSc; Nikoletta-Aikaterini Kokkali, RN; Nefeli Mavrianou-Koutsoukou, MSc; Dimitrios Patseas, MSc; and Mrs Stamatia Skourti, MSc for administrative, technical, or material support; Sentiljana Gumeni, PhD for acquisition, analysis, and interpretation of data. We also thank SYN-ENOSIS (Greece), AEGEAS (Greece), and IEMBITHEK (Greece) for partially funding this study, as well as all of the study participants for donating their time and samples.

## Disclosures

The authors have no conflicts of interest to disclose.

## References

[1] Chari A Samur MK Martinez-Lopez J. Clinical features associated with COVID-19 outcome in multiple myeloma: first results from the International Myeloma Society data set. Blood. 2020;136:3033–3040. 33367546 10.1182/blood.2020008150 PMC7759145

[2] Kastritis E Wechalekar A Schönland S. Challenges in the management of patients with systemic light chain (AL) amyloidosis during the COVID-19 pandemic. Br J Haematol. 2020;190:346–357. 32480420 10.1111/bjh.16898 PMC7300844

[3] Terpos E Trougakos IP Gavriatopoulou M. Low neutralizing antibody responses against SARS-CoV-2 in elderly myeloma patients after the first BNT162b2 vaccine dose. Blood. 2021:137:3674–3676. 33861315 10.1182/blood.2021011904 PMC8061093

[4] Herishanu Y Avivi I Aharon A. Efficacy of the BNT162b2 mRNA COVID-19 vaccine in patients with chronic lymphocytic leukemia. Blood. 2021;137:3165–3173. 33861303 10.1182/blood.2021011568 PMC8061088

[5] Tan CW Chia WN Qin X. A SARS-CoV-2 surrogate virus neutralization test based on antibody-mediated blockage of ACE2-spike protein-protein interaction. Nat Biotechnol. 2020;38:1073–1078. 32704169 10.1038/s41587-020-0631-z

[6] Walsh EE Frenck RW Jr Falsey AR. Safety and immunogenicity of two RNA-based COVID-19 vaccine candidates. N Engl J Med. 2020;383:2439–2450. 33053279 10.1056/NEJMoa2027906 PMC7583697

[7] Bird S Panopoulou A Shea RL. Response to first vaccination against SARS-CoV-2 in patients with multiple myeloma. Lancet Haematol. 2021;8:e389–e392. 33887255 10.1016/S2352-3026(21)00110-1 PMC8055205

[8] Terpos E Trougakos IP Apostolakou F. Age- and gender-dependent antibody responses against SARS-CoV-2 in health workers and octogenarians after vaccination with the BNT162b2 mRNA vaccine. Am J Hematol. 2021;96:E257–E259. 33837984 10.1002/ajh.26185 PMC8250071

